# Supplementary material for: Semiparametric mixed-effects model for analysis of non-invasive longitudinal hemodynamic responses during bone graft healing
Source: PLoS One. 2022 Apr 5;17(4):e0265471. doi: 10.1371/journal.pone.0265471 (PMC8982895; doi:10.1371/journal.pone.0265471)
Supplement: Attachment — Submitted filename: ResponseLetter_final.docx [file pone.0265471.s002.docx]

Dear Dr. Demidenko,

Thank you for handling our paper **Semiparametric Mixed-Effects Model for Analysis of Non-invasive Longitudinal Hemodynamic Responses During Bone Graft Healing** submitted to PLOS ONE. The comments and suggestions we received were very insightful. In submitting our revision, we have tried to address all the questions, which are highlighted in blue. We believe the revision has improved in quality and hope it meets your standard and can be quickly approved.

Sincerely,

Sami Leon

Jingxuan Ren

Regine Choe

Tong Tong Wu

Funding Statement:

RC and TTW's work was partly supported by grant from the National Institutes of Health NIH R01AR071363; TTW's work was also supported by grant from the National Science Foundation NSF CCF-1934962. The funders had no role in study design, data collection and analysis, decision to publish, or preparation of the manuscript.

**Response to the Editor**

Thank you for submitting your manuscript to PLOS ONE. After careful consideration, we feel that it has merit but does not fully meet PLOS ONE’s publication criteria as it currently stands. Therefore, we invite you to submit a revised version of the manuscript that addresses the points raised during the review process.

**We thank the editor for the insightful comments and suggestions. We have tried to address all the raised questions. Additionally, we reorganized the Results section to make it flow better and re-arranged the figures and tables to reduce the numbers. Below are our point-to-point responses.**

I’m not sure the authors proved that the suggested semiparametric mixed model is needed to answer the question if autograft group having higher total hemoglobin concentration than the allograft group, that is, there is a statistically significant treatment effect. The testing could be done in a much simpler and more flexible way by taking the difference between the two groups on each day and testing that the mean is zero. Either ANOVA or variance component/mixed model could applied. Note that the difference allows an arbitrary pattern of temporal changes of graft hemodynamic.

**Our aim is to evaluate the overall treatment effect and build a prediction model for the healing process. By fitting a semiparametric mixed-effects model, we are able to (1) test if there exists a significant overall treatment effect; (2) estimate the effect size of treatment; (3) model the relationship between the total hemoglobin concentration and treatment over time; (4) model the nonlinear pattern of the temporal effects; (5) make prediction at any arbitrary time point during the healing process. Being able to make prediction is important for understanding the complex nonlinear physiological responses and determining the optimal time point to differentiate between the two treatment groups with different quality of healing.**

**Obviously, ANOVA or t-test at each time point (i.e., cross-sectionally) cannot tell us if there is an overall difference in the two treatment groups. Repeated measures ANOVA can be considered as a special case of linear mixed-effects models. In this paper, we chose a semiparametric mixed-effects model by including a nonparametric function of time, f(t), to allow for greater flexibility than a linear mixed-effects model, which was used in the previous paper [18]. A linear mixed-effects model has several shortcomings: (1) the linearity assumption is too stringent and not desirable in many situations; (2) by treating the time indicator as a categorical variable, the linear mixed-effects model allows an arbitrary pattern of temporal changes. However, it is unable to make predictions at a time point different than those categorical time points (e.g., one cannot make prediction at day 2 or 3, which is not part of the time points when data were collected); (3) a linear mixed-effects model is not suitable for datasets with a large number of time points when the number of subjects is small. An additional advantage of SMM is that we could have varying time points due to adaptation made to accommodate the development of anesthesia resistance, etc. Last but not least, a SMM could also enable researchers to identify the earliest time point that begins to show a group difference. As discussed later in the Results section, we could choose Day 7 for *rTHC* and be confident the overall treatment effects would be sustained throughout the following time period. Given all these, we therefore chose a mixed-effects model with nonparametric components to accommodate the nonlinear pattern of temporal effects. The discussion was added to the last two paragraphs on page 2.**

In addition, temporal changes exhibit much variation over time and I wonder if a complex statistical model such as SLMM has grounds for this specific application.

**Thank you for pointing this out. It is obvious from the plots of the response trajectories (Figure 1) there is a nonlinear pattern, therefore nonlinear components are needed in the model. Other possible methods to incorporate the nonlinear pattern include functional data analysis. However, semiparametric mixed-effects models are a straightforward extension of linear mixed-effects models and have an easy interpretation. We agree that the variation gets larger over time in Figure 1 (b) and (c) (the variation is fairly stable in (a) and (d)), while SMM assumes a constant variance which is a limitation of the model. We are not aware of any model that can take into account the heteroscedasticity while fulfilling all of our purposes. Extending the current model by incorporating heteroscedasticity is out of the scope of this paper but will be considered in our future research. We have added the discussion in the Discussion section on page 7:**

**“The third limitation of SMM is that it cannot account for the heteroscedasticity. It is shown in Figure 1 (b) and (c) that the variation gets larger over time (whereas the variation is fairly stable in (a) and (d)), while SMM assumes a constant variance, which is a limitation. We are not aware of any model that can take into account the heteroscedasticity while fulfilling all of our purposes. Extending the current model by incorporating heteroscedasticity is out of the scope of this paper but will be considered in our future research.”**

**In addition, we realized the term “SLMM” with the “L” for linear might cause confusion. The “L” indicates the linear relationship between the response variable and the variables in the parametric part such as the treatment effect. However, it might cause confusion with the nonparametric/nonlinear part of the time effect in the model. We think “SMM” is a better abbreviation for the semiparametric mixed-effects model, therefore we used “SMM” in the revision.**

In summary, I doubt that SLMM is an appropriate model for detecting the treatment effect for these data but nevertheless I give the authors an opportunity to prove otherwise.

**We thank the editor for the opportunity to answer the questions and revise the paper. We hope our answers above are satisfactory.**
