# Supplementary material for: Semiparametric mixed-effects model for analysis of non-invasive longitudinal hemodynamic responses during bone graft healing
Source: PLoS One. 2022 Apr 5;17(4):e0265471. doi: 10.1371/journal.pone.0265471 (PMC8982895; doi:10.1371/journal.pone.0265471)
Supplement: Attachment — Submitted filename: ResponseLetter_final.docx [file pone.0265471.s003.docx]

Dear Dr. Demidenko,

Thank you for handling our revised paper **Semiparametric Mixed-Effects Model for Analysis of Non-invasive Longitudinal Hemodynamic Responses During Bone Graft Healing**. We have made all the suggested changes, including a short discussion on the overfitting issue. All the changes are highlighted in blue in the revision. We hope this revision meets your standard and can be quickly approved.

Sincerely,

Sami Leon

Jingxuan Ren

Regine Choe

Tong Tong Wu

Funding Statement:

RC and TTW's work was partly supported by grant from the National Institutes of Health NIH R01AR071363; TTW's work was also supported by grant from the National Science Foundation NSF CCF-1934962. The funders had no role in study design, data collection and analysis, decision to publish, or preparation of the manuscript.

**Response to the Editor**

Thank you for submitting your manuscript to PLOS ONE. After careful consideration, we feel that it has merit but does not fully meet PLOS ONE’s publication criteria as it currently stands. Therefore, we invite you to submit a revised version of the manuscript that addresses the points raised during the review process.

I believe one more round of revision is required: (1) although the reviewer was very positive overall his comments on the presentation are worth of consideration; (2) as I mentioned previously, semiparametric model is prone to overfitting by claiming the discovered nonlinear pattern as the pattern of noise. You have to address or at least mention this weakness in the revised version. Looking forward to receiving the updated version of your paper soon.

**We thank the editor for the helpful comments and suggestions. (1) We have incorporated all the suggested changes made by the reviewer. (2) We have also added a short discussion on the overfitting issue in the Discussion section on p.8 in the revision.**

**Response to Reviewer #1**

Reviewer #1: I am happy with the revised version, however, the paper still needs some care. I recommend acceptance subject to making the following minor corrections:

**We thank the reviewer for the insightful comments and suggestions since the first round of review. We have made all the suggested changed. Below are our point-to-point responses.**

1- The abbreviations "SMM" and "EM" are not needed in the Abstract nor the standing of LASSO

**Thanks for pointing this out. All the suggested changes were incorporated in the abstract.**

2- For the state of art in longitudinal data modeling, the authors can refer to Taavoni and Arashi (2021) and Taavoni et al. (2021).

**Thank you for providing the references. The two references have been added to the last paragraph on p.2.**

3- The first time the abbreviation SMM is defined on page 2 is sufficient. There is no need to write the whole expression further on.

**We have made the changes accordingly.**

4- The first time the nonparametric component f is defined must be pointed that it belongs to the space of polynomials.

**We have added the suggested change in the second to last paragraph on p. 3.**
